# **The DNA methylation inhibitor RG108 protects against noise-induced hearing loss**

**DOI:** 10.1007/s10565-021-09596-y

**Published:** 2021-03-15

**Authors:** Zhiwei Zheng, Shan Zeng, Chang Liu, Wen Li, Liping Zhao, Chengfu Cai, Guohui Nie, Yingzi He

**Affiliations:** 1 ENT institute and Department of Otorhinolaryngology, Eye & ENT Hospital, Fudan University, 83 Fenyang Road, Shanghai, 200031 China; 2 NHC Key Laboratory of Hearing Medicine (Fudan University), Shanghai, 200031 China; 3 Department of Otorhinolaryngology Head and Neck Surgery, The First Affiliated Hospital, School of Medicine, Xiamen University, Xiamen, 361003 People’s Republic of China; 4 Department of Otolaryngology and Institute of Translational Medicine, Shenzhen Second People’s Hospital/the First Affiliated Hospital of Shenzhen University Health Science Center, Shenzhen, 518035 China

**Keywords:** Noise-induced hearing loss, Cochlea, Hair cells, DNA methylation

## Abstract

**Background:**

Noise-induced hearing loss represents a commonly diagnosed type of hearing disability, severely impacting the quality of life of individuals. The current work is aimed at assessing the effects of DNA methylation on noise-induced hearing loss.

**Methods:**

Blocking DNA methyltransferase 1 (DNMT1) activity with a selective inhibitor RG108 or silencing DNMT1 with siRNA was used in this study. Auditory brainstem responses were measured at baseline and 2 days after trauma in mice to assess auditory functions. Whole-mount immunofluorescent staining and confocal microcopy of mouse inner ear specimens were performed to analyze noise-induced damage in cochleae and the auditory nerve at 2 days after noise exposure.

**Results:**

The results showed that noise exposure caused threshold elevation of auditory brainstem responses and cochlear hair cell loss. Whole-mount cochlea staining revealed a reduction in the density of auditory ribbon synapses between inner hair cells and spiral ganglion neurons. Inhibition of DNA methyltransferase activity via a non-nucleoside specific pharmacological inhibitor, RG108, or silencing of DNA methyltransferase-1 with siRNA significantly attenuated ABR threshold elevation, hair cell damage, and the loss of auditory synapses.

**Conclusions:**

This study suggests that inhibition of DNMT1 ameliorates noise-induced hearing loss and indicates that DNMT1 may be a promising therapeutic target.

**Graphical abstract:**

**Figure:**
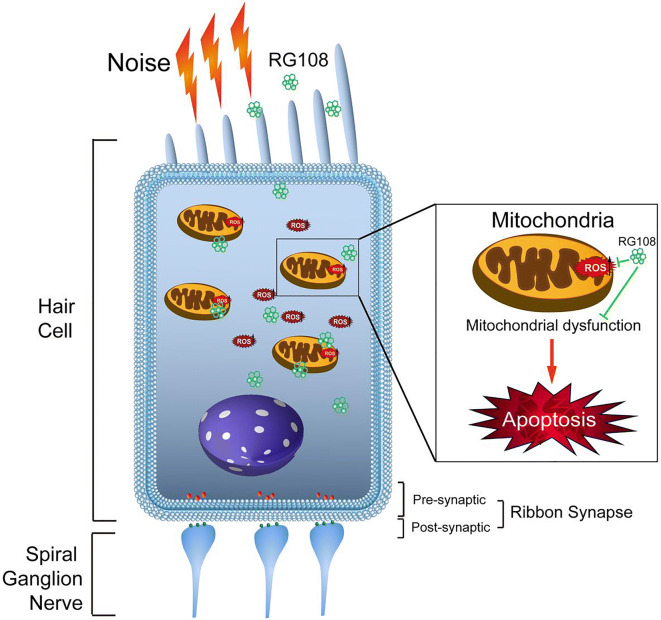
**Graphical Headlights** • RG108 protected against noise-induced hearing loss • RG108 administration protected against noise-induced hair cell loss and auditory neural damage. • RG108 administration attenuated oxidative stress-induced DNA damage and subsequent apoptosis-mediated cell loss in the cochlea after noise exposure.

**Supplementary Information:**

The online version contains supplementary material available at 10.1007/s10565-021-09596-y.

## Background

Noise-induced hearing loss (NIHL) represents an important health challenge significantly affecting the quality of life of individuals and imposing a substantial burden on the society. To date, no clinically applicable therapies are available to prevent or mitigate the pathology of NIHL (Mukherjea et al. 2015), due to the complex underlying cellular and molecular mechanisms (Kobel et al. 2017; Sha and Schacht 2017). Permanent NIHL is due to loss of cochlear hair cells (HCs), particularly outer hair cells (OHCs) (Kurabi et al. 2017; Le Prell et al. 2007; Sha and Schacht 2017). Sensory HCs in the inner ear normally convert mechanical sound stimuli into receptor potentials, and HC damage is the primary event in hearing loss. Excessive noise results in irreversible damage to inner ear sensory HCs and induces irreversible hearing loss, because cochlear HCs are unable to spontaneously regenerate in adult mammalian organisms. Besides, recent evidence clearly shows that cochlear ribbon synapse, formed between inner hair cells (IHCs) and spiral ganglion neuron (SGN) terminals, is essential for normal hearing, and its degeneration is another key contributor in NIHL, leading to the degeneration of SGNs and permanent threshold shift (Furman et al. 2013; Kujawa and Liberman 2009; Lin et al. 2011). Thus, therapeutic strategies for appropriate protection against noise-induced hearing loss should address both cochlear HC injury and synaptopathy.

Numerous studies have shown that many parameters contribute to NIHL pathogenesis, e.g., intracellular Ca^2+^ overload, ATP depletion, and excessive oxidative stress (Hu et al. 2008; Kurabi et al. 2017; Sha and Schacht 2017). Oxidative damage in the cochlea caused by overproduction of free radicals, including reactive oxygen (ROS) and nitrogen (RNS) species, constitutes an important mechanism of NIHL (Fetoni et al. 2013; Ohinata et al. 2003). The markers of oxidative stress 4-hydroxynonenal (4-HNE) and 3-nitrotyrosine (3-NT) show elevated amounts in sensory HCs upon challenge with noise (Yuan et al. 2015b). High ROS and RNS amounts could damage all cell components (proteins, lipids, carbohydrates, and DNA); in addition, they affect genes involved in apoptotic pathways, resulting in apoptosis of cochlea HCs and leading to irreversible hearing loss (Hu et al. 2008; Kurabi et al. 2017; Yuan et al. 2015b). Multiple studies assessing interventions for NIHL have focused on discovering and developing new antioxidant compounds for improved prevention and/or treatment of HC damage and auditory nerve fiber degeneration induced by noise exposure; however, most reported drug candidates are partially effective in NIHL prevention or alleviation (Harris et al. 2006; Le Prell et al. 2007; Shi et al. 2007). Thus, several new and epigenetic mechanisms of transcriptional regulation have been highlighted that might influence the extent of damage from NIHL (Chen et al. 2015; Hill et al. 2016).

Epigenetic mechanisms of transcriptional modulation, such as DNA methylation and histone modifications, have recently been implicated in various biological processes and disease progression [6]. Several studies have demonstrated that epigenetic events, such as histone modifications, occur in the cochlea of mice exposed to noise; therefore, histone deacetylase inhibitors could be effective otoprotectants against noise-induced HC loss and hearing damage based on their antioxidant effects by modulating gene expression (Chen et al. 2016; Wen et al. 2015). These findings indicate that epigenetic regulations have both therapeutic and prophylactic effects in NIHL. DNA methylation represents a covalent DNA alteration catalyzed by the DNA methyltransferase (DNMTs) family. It is involved in multiple biological events such as regenerative capacity (Yakushiji et al. 2009) and stem-cell fate (Gereige and Mikkola 2009). Aberrant DNA methylation is closely associated with many diseases such as cancer. Currently, a great deal of attention is being paid to the potential role of DNA methylation in stem cell fate determination (Deng et al. 2019). DNMT inhibitors have been shown to modulate methylation in the entire genome of mouse utricle sensory epithelia-derived progenitor cells (MUCs) for upregulating sensory HC genes and proteins, indicating that DNA methylation may be also a critical mechanism underpinning epigenetic modification-dependent gene transcription in HCs (Deng et al. 2019).

Recent studies have shown that RG108, a novel non-nucleoside small-molecule DNMT inhibitor designed to target human DNA methyltransferase-1 (DNMT1), interacts with the catalytic domain of this enzyme and blocks its active site with low genotoxicity and cytotoxicity (Lyko and Brown 2005; Siedlecki et al. 2006). Several studies have used RG108 for investigating the epigenetic mechanisms involved in physiological conditions and several human disorders, including cancers and neurological diseases (Graca et al. 2014; Oh et al. 2015). Oh et al. suggested that RG108 could alleviate cellular damage due to oxidative stress by restoring the altered methylation pattern of senescence-associated genes, which provides improved treatment efficacy in aging-related diseases (Oh et al. 2015). It was recently reported that RG108 combined with histone deacetylase inhibitor protects retinal pigment epithelial cells from the detrimental effects of oxidative stress by inducing antioxidative enzyme gene expression (Tokarz et al. 2016). In addition to its role as an antioxidant, RG108 can also regulate the whole family of genes that encode ion channels. DNA methyltransferase inactivation by RG108 increases the inherent membrane excitability of cortical pyramidal neurons *in vitro*, evidenced by elevated evoked AP frequency (Meadows et al. 2016). The confirmed ability and the lack of cytotoxicity make RG108 a promising new candidate for epigenetic therapies. Thus, this work is aimed at testing the hypothesis that RG108 has therapeutic effects in NIHL by targeting at multiple points of vulnerability in the inner ear, including HC injury and ribbon synaptic loss.

## Methods

### Animals

C57BL/6J mice (12 weeks old) were chosen for this study, because this mouse strain shows an excellent susceptibility to NIHL (Brown et al. 2014; Yan et al. 2013). Experiments involving animals had approval from the Institutional Animal Care and Use Committee of Fudan University.

### RG108 administration via the intraperitoneal route

RG108 (Selleck Chemicals, USA; S2821) at 200 mg/mL in dimethyl sulfoxide (DMSO) was kept at −20°C. This stock solution underwent further dilution with 0.9% saline right before injection. Animals were intraperitoneally administered RG108 at 1 or 10 mg/kg once, 2 h before noise exposure.

### DNMT1 siRNA delivery

DNMT1 siRNA (siDNMT1; MISSION siRNA ID: SASI_Mm01_00024007; Sigma, USA) or scrambled siRNA (siControl; MISSION siRNA Universal Negative Control; Sigma) was locally delivered via post-auricular microinjection via the round window membrane. First, siDNMT1 and siControl each at 0.3 and 0.6 μg were assessed in preliminary assays, and 0.6 μg showed relatively high knockdown efficiency. A previously described protocol was utilized for inner ear siRNA delivery (Oishi et al. 2013), with minor modifications. Briefly, after anesthesia (IP administration of ketamine and xylazine at 100 and 20 mg/kg, respectively), the retro-auricular surgical method was applied to approach the temporal bone. Next, a shallow hole was generated in the otic bulla with a 30-G needle and enlarged to 2 mm in diameter for visualizing the round window. This was followed by the insertion of a micro-medical tube into the hole, with the tube suspended above the round window for slow delivery of 0.6 μg (10 μl) of DNMT1 siRNA or siControl to fill the round window niche. Based on previous studies, siRNA administration to animals causes a transient increase of auditory threshold, which completely recovers by 72 h. Therefore, noise exposure was carried out 72 h following siRNA administration.

### Noise exposure

Noise exposure was carried out according to a previous report (Yuan et al. 2015a). Briefly, the noise signal was generated by a System III processor from Tucker-Davis-Technologies (TDT, Alachua, FL, USA) and amplified by a Yamaha P9500S power amplifier. Awake two-month-old mice were exposed to acute open-field white noise for 2 h at 120 dB SPL. Sound levels for noise exposure are calibrated with a sound level meter at multiple locations within the sound chamber to ensure uniformity of the sound field and are measured before and after exposure to ensure stability. The variation in sound level across the cage was less than 1 dB. Control mice were subjected to an identical procedure except the noise was not turned on (sham exposure).

### Auditory brainstem response (ABR) measurements

The mice underwent anesthesia as described above and kept warm in the course of ABR recordings. The TDT BioRigRZ system (Tucker-Davis Technologies, USA) was utilized for ABR measurements, as directed by the manufacturer. Briefly, ABR potentials were evoked with 5 ms (4 ms and 1-ms rise–fall) tone pips administered to the left eardrum at 4, 8, 16, 24, and 32 kHz utilizing a metal loudspeaker in the external auditory meatus. The minimal stimulus levels producing reproducible responses for ABR wave II in various animals were determined as the ABR thresholds. The amplitude was assessed as the peak-to-peak (p-p) amplitude difference between wave I peak and its trough. Latency was reflected by the time from stimulus onset to wave I peak. Measurements were made at all above frequencies.

### Immunohistochemistry

Cochlear specimens underwent fixation at 4°C with 4% formalin in 0.01 M PBS (pH 7.4) overnight and decalcification with 10% ethylenediamine tetra-acetate (EDTA) for 72 h at 4°C. The remaining cochlear sensory epithelium was divided into the apex, middle, and base segments, which underwent blocking with 10% normal goat serum in 0.01 M PBS for 1 h at ambient. Next, the segments underwent overnight incubation at 4°C with the following primary antibodies: anti-myosin 7a (1:500, Proteus Biosciences, 25-6790), anti-nitrotyrosine (1:200, EMD Millipore, USA, 05-233), anti-CtBP2 IgG1 (1:500, BD Biosciences, 612044), anti-GluR2 IgG2a (1:1000, EMD Millipore, MAB397), anti-DNMT1 (1:500, Abcam, UK, ab13537), and anti-5-mC (1:500, Abcam, ab10805). This was followed by PBS washes and incubation with secondary antibodies (Invitrogen, 1:1000) at 4°C overnight away from light. The TCS SP8 laser scanning confocal microscope (Leica, Germany) was utilized for image acquisition and analysis.

### Immunohistochemistry for cochlear cryosections

Cochlear specimens after PBS washes were decalcified with 10% EDTA at 4°C with shaking and successively added to 15% and 30% sucrose in PBS for 30 min (at ambient) and 4°C (overnight), respectively. Then, optimal cutting temperature compound embedding was performed, followed by cutting into 10-12 μm mid-modiolar sections and mounting on glass slides. Sections underwent blocking with 10% normal goat serum (1 h at ambient) and incubation with primary antibodies, including anti-myosin 7a (1:500, Proteus Biosciences, 25-6790), anti-Sox2 (1:200, Santa Cruz, USA, sc-17320), anti-neurofilament (1:500, Abcam, ab72996), and anti-Tuj-1 (1:200, BioLegend, USA; 801202) at 4°C for 48 h. After three washes, incubation was carried out with secondary antibodies (1:200) at 4°C overnight away from light.

### Phalloidin staining

Fixed and permeabilized specimens underwent incubation with Alexa Fluor 488- or 647-conjugated phalloidin (1:1000; Invitrogen) for 30 min away from light and DAPI counterstaining for 10 min, for visualizing hair cell F-actin and nuclei, respectively.

### TUNEL assay

To evaluate cell apoptosis, the TUNEL (terminal deoxynucleotidyl transferase-mediated dUTP nick-end labeling) assay was carried out with a kit from Roche (USA; Cat. no.11684795910), as directed by the manufacturer. In brief, inner ear sections or cochlear specimens underwent three PBST washes of 10 min each. Then, cochlear specimens underwent staining with TUNEL working solution at 37°C for 1 h away from light.

### Caspase-mediated apoptosis assay

Cochlear specimens after PBS washes underwent staining with 5 μM Caspase 3/7 Green Detection Reagent (Life Technologies, C10723) away from light for 10 min at **37**°C. The Leica SP8 confocal fluorescence microscope was utilized for data analysis**.**

### ROS detection

Cochlear specimens upon PBS washes underwent staining with 5 μM MitoSox-red (Life Technologies, 1771410) as directed by the manufacturer. The Leica SP8 confocal fluorescence microscope was utilized to acquire fluorescent images**.**

### Assessment of IHCs and OHCs

Perfectly shaped hair cells with intact nuclei and myosin 7a staining in confocal images were considered surviving hair cells and quantified with Image J (National Institutes of Health). Average numbers of IHCs and OHCs per 200 μm of basal, middle, and apical cochlear segments were obtained, respectively.

### Quantitation of cochlear ribbon synapses

To quantify the ribbon synapses of IHCs, cochlear samples stained with anti-CtBP2 antibodies were used. Functional ribbon synapses were counted manually by visualization of GluR2 colocalization with CtBP2 in IHCs with Image J.

### Quantitation of SGNs

For SGN quantitation, Tuj-1 labeled somatic cells with large round nuclei within the Rosenthal’s canal were counted from the maximum intensity projection in each section, and neuronal density was expressed as SGN density (per 10,000 μm^2^).

### Data analysis

Data are mean ± SEM and were assessed with GraphPad Prism 7 (GraphPad Software Inc., USA). One-way ANOVA, two-way ANOVA, or unpaired Student’s t-test was performed for comparisons. Two tailed *p*<0.05 indicated statistical significance.

## Results

### RG108 attenuates noise-induced auditory threshold shifts

**Fig. 1:**
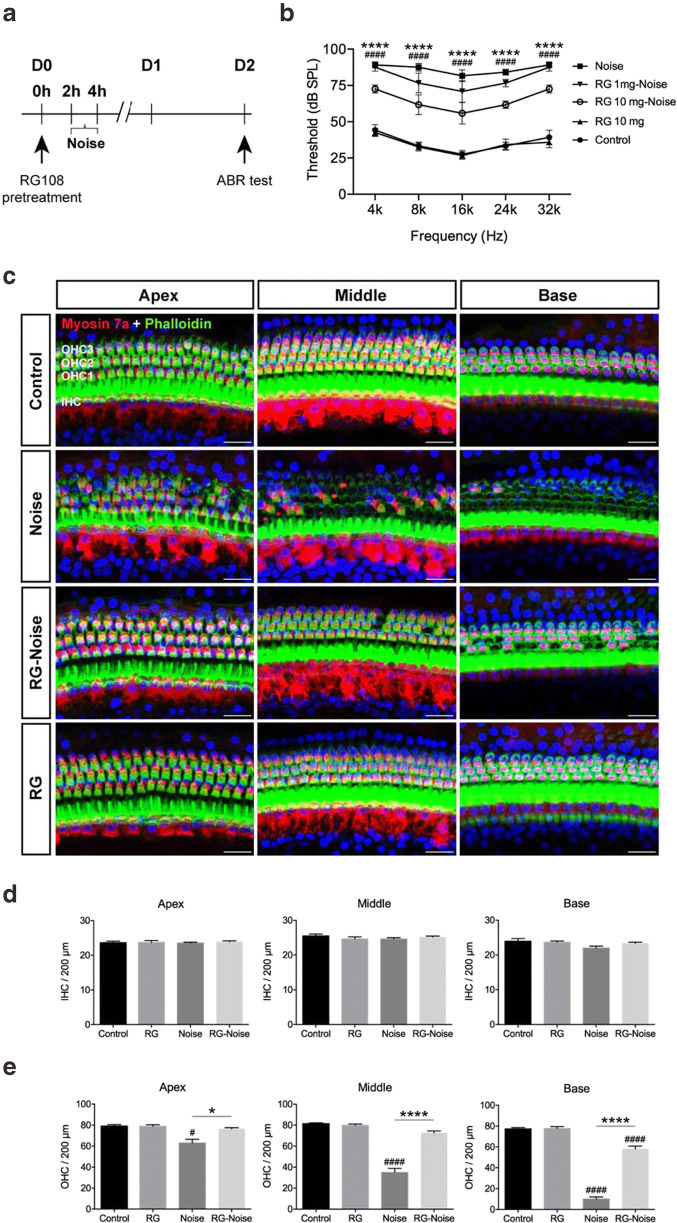
Assessment of ABR threshold shifts and hair cell (HC) loss at 2 days after noise exposure. (a) Experimental schedule. Mice pre-injected with saline or RG108 were exposed to 120-dB noise for 2 h, and the ABR thresholds were measured at 4, 8, 16, 24, and 32 kHz at 2 days after noise exposure. (b) Noise exposure caused elevation of ABR thresholds across tested frequencies, while pretreatment with 10 mg RG108 significantly attenuated the noise-induced ABR threshold shifts at each tested frequency. *n* = 6 mice for each condition. Data are mean ± SEM. Statistical analysis was performed using a two-way ANOVA, ^####^*p* < 0.0001 for noise vs. control, ****p < 0.0001 for noise vs. 10 mg RG108-Noise. (c) Representative images of myosin 7a and phalloidin staining of cochlear turns from different groups at 2 days after noise exposure. OHC1, OHC2, OHC3: 1st, 2nd, and 3rd rows of outer hair cells (OHCs); inner hair cell (IHC). Scale bar = 20 μm. (d, e) Quantitative analysis of noise-induced losses of IHC (d) and OHC (e) along the cochlear explants. Total IHC or OHC count per 200 μm was compared among no noise (control), 10 mg RG108 (RG), noise, and 10 mg RG108-Noise (RG-Noise) groups. A significant loss of OHC was observed at both base and middle turns in noise-exposed mice compared with no noise control mice. In contrast, OHC loss in the RG108 pretreatment group was significantly lower than that in the noise exposure group, but still higher than in the no noise control group (except middle and apex turns). No significant differences were observed in IHCs loss among these groups. *n* = 6 mice for each condition. Data are mean ± SEM. Statistical analysis was performed using a one-way ANOVA, ^#^*p* < 0.05, ^####^*p* < 0.0001 for noise vs. control; *p < 0.05 and ****p < 0.0001 for noise vs. RG-Noise. IHC, inner hair cell; OHCs, outer hair cells

In this study, RG108’s effects on NISH were determined. Adult mice were randomized to the control (no noise exposure; normal controls), noise (noise exposure without RG108 treatment), and RG108-noise (both noise exposure and RG108 administration) groups. Mice in the RG108-noise group were administered a single i.p. dose (1 mg/kg or 10 mg/kg) of RG108 2 h before noise exposure. The experimental schedule is described in Fig. 1a. The mice evaluated in these experiments received ABR measurements at varying frequencies of 4, 8, 16, 24, and 32 kHz, respectively, to reflect susceptibility to NIHL. As shown in Fig. 1b, hearing thresholds were significantly higher at 2 days after noise exposure compared with control values at all frequencies (*n*=6, two-way ANOVA, *p*<0.0001, Fig. 1b). Treatment with 1 mg/kg RG108 reduced auditory threshold shifts by ~ 10 dB at 8 (*p*<0.0001), 16 (*p*<0.0001), and 24 (*p*<0.05) kHz, respectively, but not at 4 and 32 kHz, in comparison to the noise group. Following RG108 administration at 10 mg/kg, noise-induced auditory threshold shifts at each frequency tested were significantly reduced compared with the noise group (*n*=6, two-way ANOVA, *p*<0.0001, Fig. 1b). We therefore selected the 10 mg/kg dose for RG108 administration based on the dose response, for subsequent experiments assessing its protective effects in NIHL.

### RG108 protects against noise-induced HC loss

**Fig. 2:**
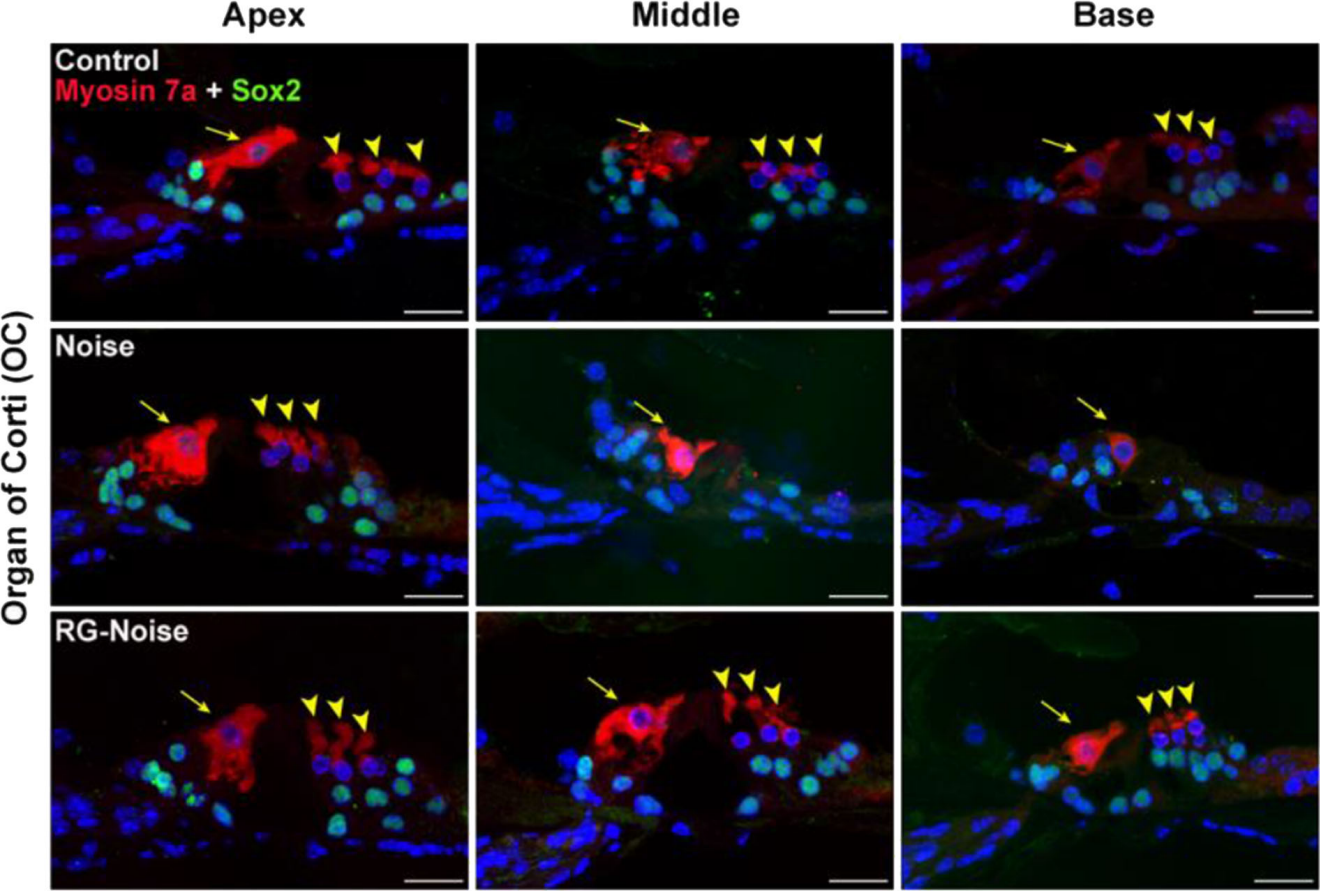
Assessment of hair cell (HC) loss at 2 days after noise exposure. Immunostaining was performed in the inner ear sections of mice from different groups. Representative images showed reduction in immunoreactivity for myosin 7a (red) and Sox2 (supporting cell marker, green) in the organ of Corti (OC) after noise exposure compared to controls without exposure. Pretreatment with RG108 significantly attenuated noise-induced decrease of OHCs and supporting cells. Images were taken from the apical, middle, and basal turns of cochlea in the inner ear sections. The yellow arrowheads point to three rows of outer hair cells, and the yellow arrow indicates an inner hair cell. Scale bar =20 μm

To identify potential causes of noise-induced hearing loss, HCs were stained with anti-myosin 7a antibodies to assess noise-associated HC loss as well as the protective effects of RG108. Noise exposure induced a dramatic loss of outer hair cells (OHCs) following a base-to-apex gradient (Fig. 1c). In accordance with decreased myosin 7a immunolabeling of HCs, Alexa Fluor 488 phalloidin immunostaining of the actin cytoskeleton revealed scarred areas on cochlear surface preparations from base to apex (Fig. 1c). In contrast, myosin 7a immunostaining and quantitative analysis showed that OHC counts were robustly elevated in the RG108-noise group compared with the noise group (Fig, 1c). These data are summarized in Fig. 1d, e. This finding was confirmed by immunofluorescent staining of cochlear sections. Noise exposure significantly decreased the amounts of OHCs at both basal and middle cochlear segments compared with unexposed controls, while pretreatment with RG108 substantially protected OHCs from noise-associated damage (Fig. 2).

### RG108 reduces noise-induced auditory neural damage

**Fig. 3:**
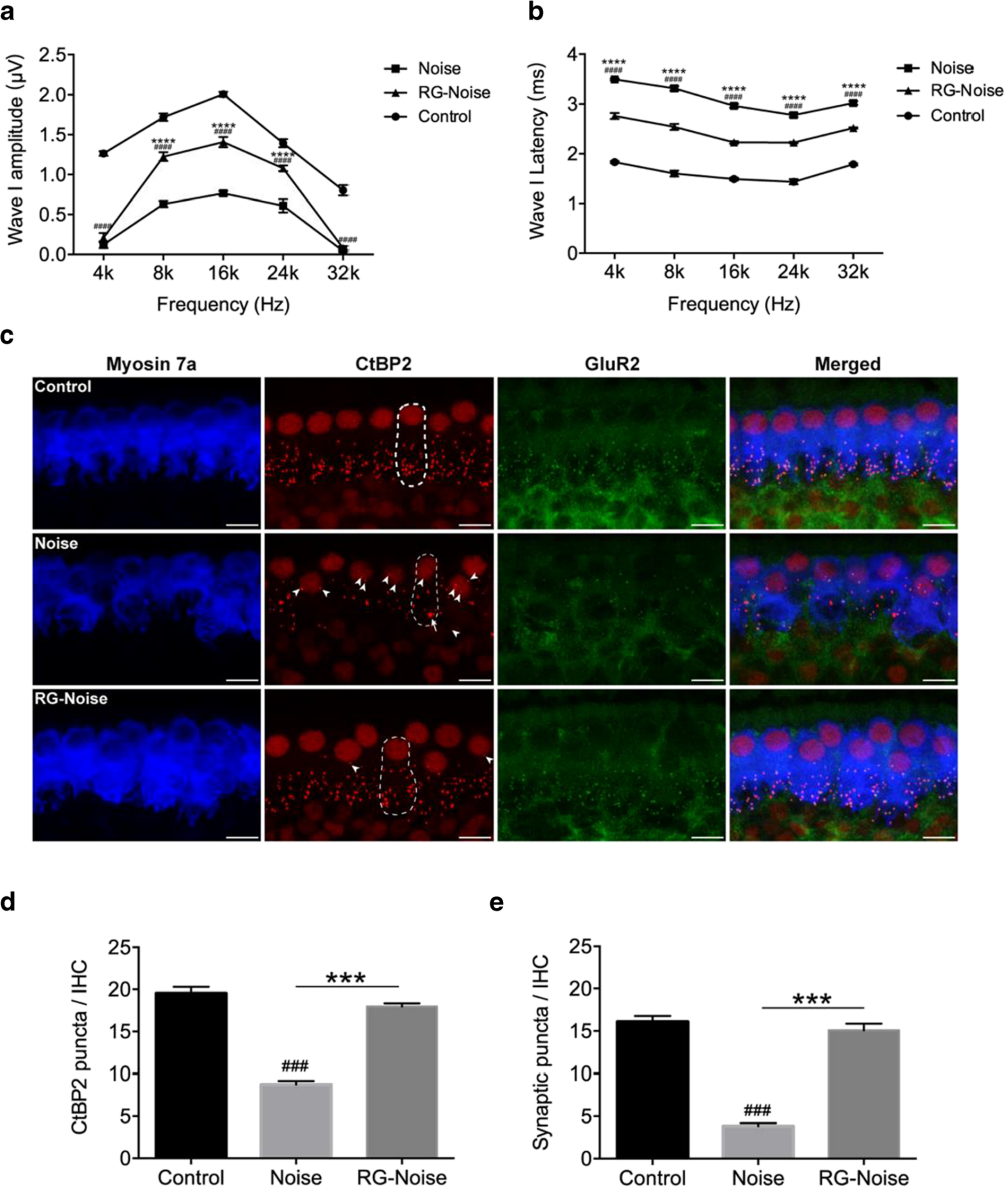
Assessment of noise-induced losses of synapses. (a, b) Representative ABR wave I amplitudes and latencies at 4, 8, 16, 24, and 32 kHz in mice for each condition. ABR wave I amplitudes in RG108 pretreated mice were significantly increased compared to the noise exposed mice. Threshold adjusted ABR wave I latency measurements demonstrated that RG108 pretreatment prevented increase of wave I latency induced by noise when tested 2 days after the noise exposure. *n* = 6 mice for each condition. Data are mean ± SEM. Statistical analysis was performed using a two-way ANOVA, ^####^*p* < 0.0001 for noise vs. control, ****p < 0.0001 for noise vs. RG-Noise. (c) Representative confocal images of immunolabeling for presynaptic ribbons (CtBP2-red), postsynaptic receptor patches (GluR2-green), and IHCs (myosin 7a-blue) in the middle turn corresponding to 16 kHz in each group (no noise-exposed cochlea; noise-exposed cochlea; RG108-treated cochlea at 2 days post-noise exposure). The white dash lines indicate the outline of IHCs. The CtBP2 antibody also stains hair cell nuclei. The white arrowheads point to ribbons that are far from the basolateral surface of the IHC toward the perinuclear positions. The white arrow indicates abnormally large ribbons in the exposed ear. Scale bar = 10 μm. (d, e) Quantitative data showed that noise reduced, and RG108 pretreatment increased, the number of CtBP2-immunolabeled ribbons (d) and paired ribbon puncta, defined as juxtaposed CtBP2- and GluR2-positive puncta (e) at 16 kHz cochlear region; *n* = 24 IHCs from four cochleae of each group. Data are mean ± SEM. ^###^*p* < 0.001 for noise vs. control; ***p < 0.001 for noise vs. RG108-Noise

To further assess RG108’s effects in NIHL, we measured ABR wave I amplitudes and latencies in the normal control, noise, and RG108-noise groups (Fig. 3a, b). As shown in Fig. 3a, noise exposure remarkably reduced wave I amplitudes compared with normal controls, whereas RG108 administration starkly alleviated this noise-associated decrease (Fig. 3a). ABR wave I latencies are presented in Fig. 3b. ABR wave I latencies at 4, 8, 16, 24, and 32 kHz were overtly enhanced in noise-exposed animals in comparison with unexposed control mice. Meanwhile, ABR latencies were significantly reduced in RG108 pretreated mice compared with noise-exposed animals. Since the wave I amplitude and latency reflect all the activity of the remaining auditory nerve fibers, we wondered if these changes in the noise-exposed group were, at least partly, due to the degeneration of ribbon synapses between IHCs and auditory nerve fibers of spiral ganglion cells.

As pre-synaptic ribbon amounts provide an informative measure of IHC efferent innervation, we immunostained synapses with an antibody targeting the major presynaptic ribbon protein C-terminal binding protein 2 (CtBP2), with specific staining of both presynaptic ribbons and nuclei. As shown in epithelial whole mounts imaged in Fig. 3c, anti-CtBP2 labeling in unexposed controls displayed small puncta arrayed in the subnuclear area, around the basolateral IHC membrane. After noise exposure, confocal analysis revealed a significant reduction of pre-synaptic ribbons throughout the cochlea; in addition, the amounts of CtBP2 spots were reduced compared with the no-noise condition, while pretreatment with RG108 significantly protected ribbons from noise-induced damage (Fig. 3c, d). Next, we compared the numbers of functional synapses reflected by juxtaposed presynaptic ribbons (small red dots; stained with CtBP2) and postsynaptic glutamate receptors (green dots; stained with GluR2) 2 days after noise exposure. In unexposed controls, immunostaining revealed juxtaposed CtBP2 and GluR2 studding the bottom of IHCs. Compared with unexposed controls, a severe decrease in CtBP2 and GluR2 density was evident in noise-treated mice as measured at the area corresponding to 16 kHz. By contrast, comparison of both noise-exposed groups showed that pretreatment with RG108 significantly reduced noise-induced loss of synapses (Fig. 3c, e).

**Fig. 4:**
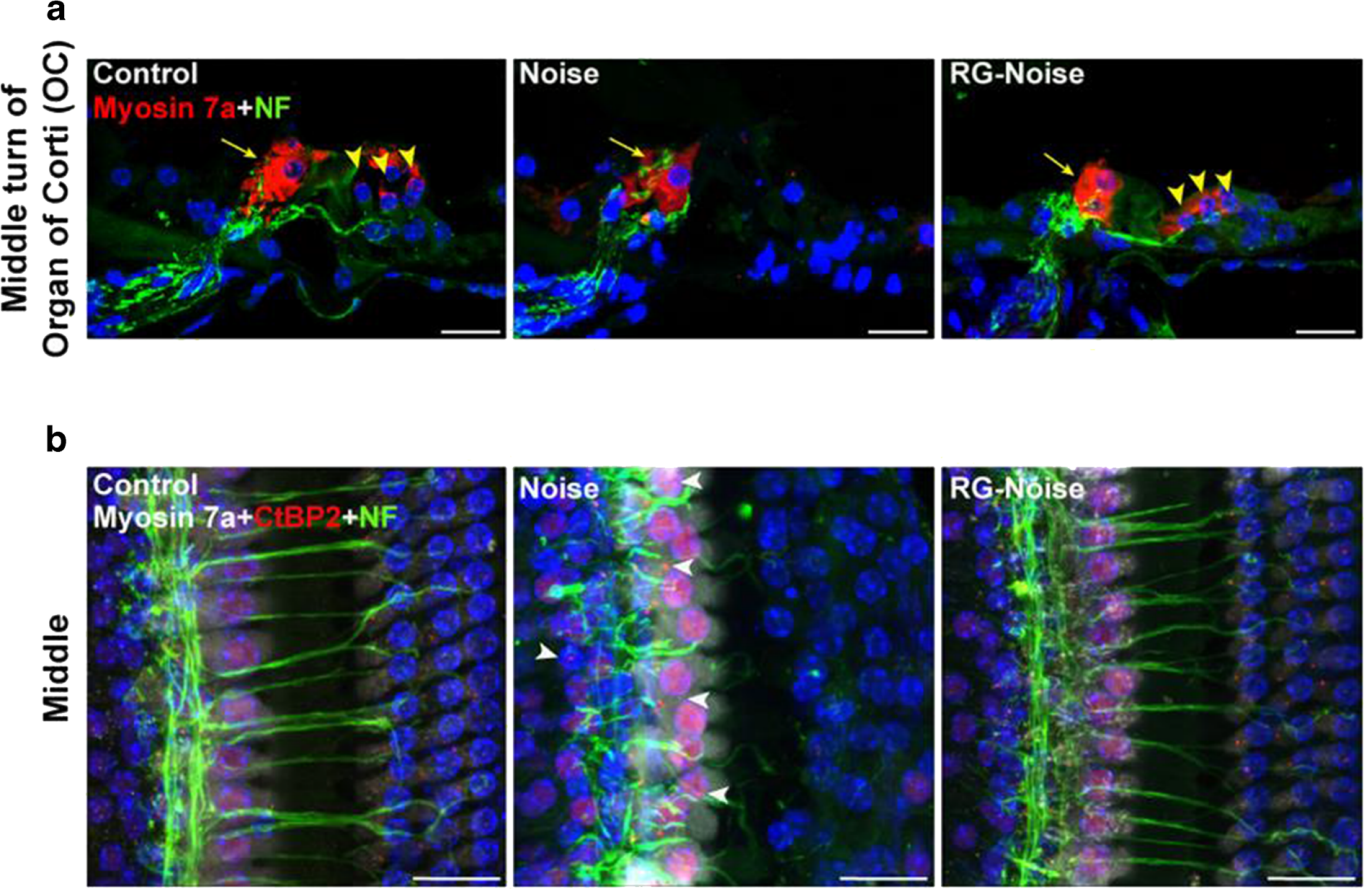
Assessment of noise-induced synaptic degeneration. (a) Analysis for cochlear nerve terminals (neurofilament, NF-green) and HCs (myosin 7a-red) in mice for each condition. Images were taken from the middle turn of cochlea in the inner ear sections. The yellow arrowheads point to three rows of outer hair cells, and the yellow arrow indicates an inner hair cell. Scale bar = 20 μm. (b) Assessment of synaptic ribbons (CtBP2-red), cochlear nerve terminals (neurofilament, NF-green), and HCs (myosin 7a-white) in mice for each condition. Images were obtained from the middle cochlear turn. The white arrowheads in noise image point to ribbons are not paired with auditory nerve terminals, and some appear far from the basolateral surface of the IHC. Scale bar = 20 μm

Confocal microscopy showed that some remaining ribbons in the noise-exposed group were abnormally larger. In addition, some ribbons were far from the basolateral surface of IHCs toward the perinuclear positions or even above the nuclei in IHCs, but not found in these positions in normal controls (Fig. 3c). Therefore, we assessed whether the migrated ribbons were no longer involved in synaptic transmission. To this end, we assessed ribbons and auditory nerve terminals by staining for CtBP2 and neurofilament (NF). The results showed that all unmyelinated cochlear nerve fiber terminals were under the IHCs. In normal unexposed controls, all ribbons were surrounded the terminals of the auditory nerve; in contrast, after noise exposure, the migrated perinuclear ribbons were displaced away from the closest auditory nerve terminals (Fig. 4).

As disruption of spiral ganglion neurons (SGNs) is considered to cause hearing loss, we then assessed SGN survival in controls without noise exposure and NIHL mice treated with or without RG108. In this study, SGNs in cochlear sections were quantified by Tuj-1-labeled cell bodies and DAPI-labeled large round cell nuclei (Supplementary Figure S1). Quantitative analysis showed that the numbers of Tuj-1-positive neurons in spiral ganglions of normal controls, noise-exposed controls, and RG108 pretreated mice at 2 days after noise exposure were comparable. These results demonstrated that neuronal loss, previously considered to be associated with hair cell death, was not obviously detectable at the time points of the current experiments.

### RG108 decreases oxidative DNA damage and cell apoptosis following noise exposure

**Fig. 5:**
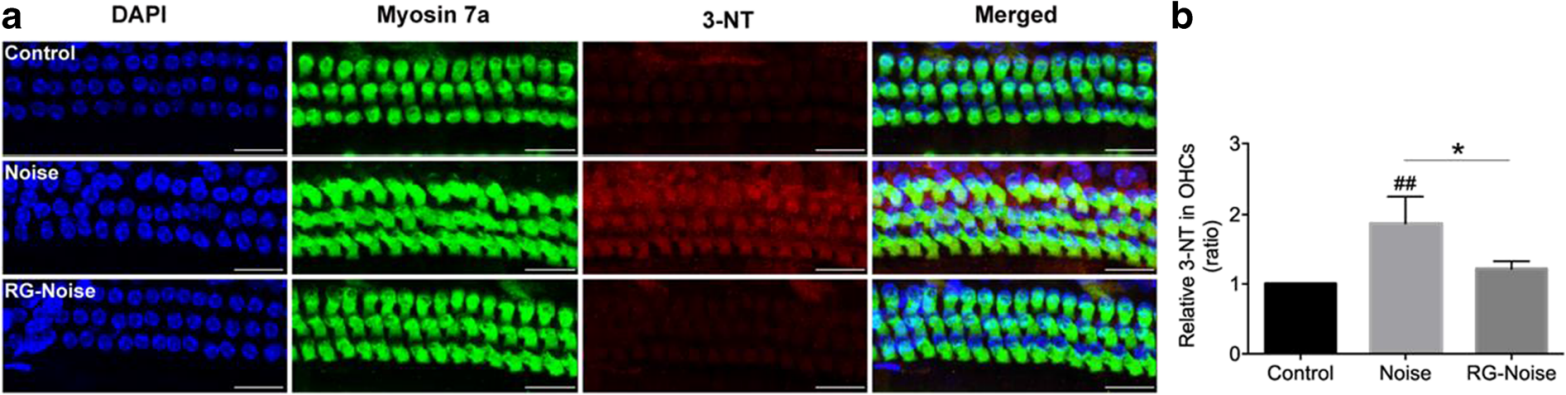
Immunofluorescence analyses of oxidative stress-associated 3-nitrotyrosine (3-NT) expression in OHCs. (a) Representative images of myosin 7a (green) and 3-NT (red) staining of apical cochlear turns from different groups at 2 days after noise exposure. RG108 treatment reduced the noise-increased 3-NT level in OHCs. Scale bar = 20 μm. (b) Quantification of 3-NT staining in OHCs confirmed a significant reduction with RG108 administration. *n* = 3 per group with one cochlea used per mouse. Data are mean ± SEM. ^##^*p* < 0.01 for noise vs. control; *p < 0.05 for noise vs. RG-Noise

**Fig. 6:**
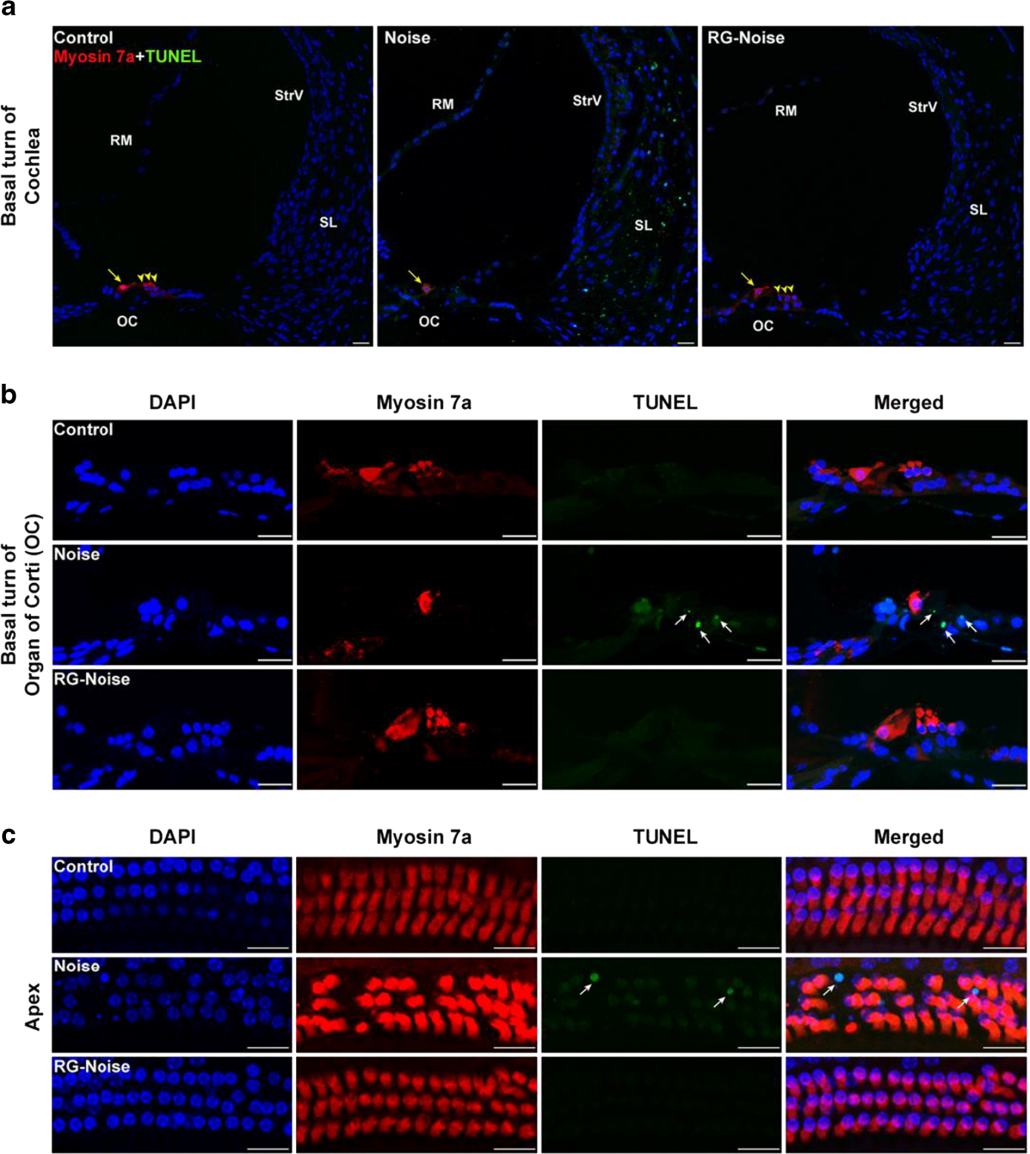
Detection of apoptotic cell death by TUNEL assay in the cochlea. (a, b) Immunostaining was performed in the inner ear sections of mice from different groups. Representative images showed an increase in immunoreactivity for TUNEL (green) in the spiral ligament (SL) and organ of Corti (OC) after noise exposure compared to controls without exposure. RG108 pretreatment significantly attenuated noise-induced apoptosis. Images were obtained from the basal turn of cochlea. The yellow arrowheads in (a) point to three rows of outer hair cells, and the yellow arrow indicates an inner hair cell. The white arrows in (b) point to TUNEL-positive cells. Scale bar = 20 μm. (c) Representative images of TUNEL staining of cochlea from different groups at 2 days after noise exposure. Images were obtained from the apical cochlear region. Scale bar = 20 μm. OC: organ of Corti; RM: Reissner’s membrane; SL: spiral ligament; StrV: stria vascularis

Given that oxidative damage is a critical factor in NIHL-induced cochlear cell death (Fetoni et al. 2013), we formulated the hypothesis that RG108 might attenuate oxidative stress-induced DNA damage and the resulting apoptosis-associated cell loss in the cochlea upon noise exposure, protecting from NIHL. To address this research question, staining of protein 3-nitrotyrosine (3-NT), an effective biomarker of oxidative DNA damage (Yamashita et al. 2004), was carried out to assess the whole cochlear tissue from control conditions without noise exposure and NIHL animals administered RG108 or not. We found that 3-NT was significantly elevated in OHCs 2 days upon noise exposure in comparison with controls without noise exposure (Fig. 5). However, RG108 pretreatment starkly alleviated this effect (Fig. 5), suggesting that RG108 indeed reduced oxidative damage to cochlear sensory cells. Oxidative damage is known to trigger cell death mainly through apoptosis (Fetoni et al. 2013; Oishi and Schacht 2011). We next asked whether RG108 decreases apoptosis induced by noise exposure in the cochlea, as measured by TUNEL staining of cochlear sections from normal controls, noise-exposed controls, and RG108 pretreated mice, respectively (Fig. 6). After noise exposure, abundant green fluorescent signals, indicating TUNEL-positive cells, were recorded in the spiral ligament (SL) (Fig. 6a), and some positive cells were found in the organ of Corti (OC) (Fig. 6b). Corroborating oxidative damage attenuation by RG108, pretreatment with RG108 significantly reduced the amounts of TUNEL-positive cells in the respective cochlear areas mentioned above (Fig. 6a–c). Taken together, these data indicated that RG108 could significantly suppress noise-induced apoptosis.

### DNMT1 siRNA treatment alleviates NIHL

**Fig. 7:**
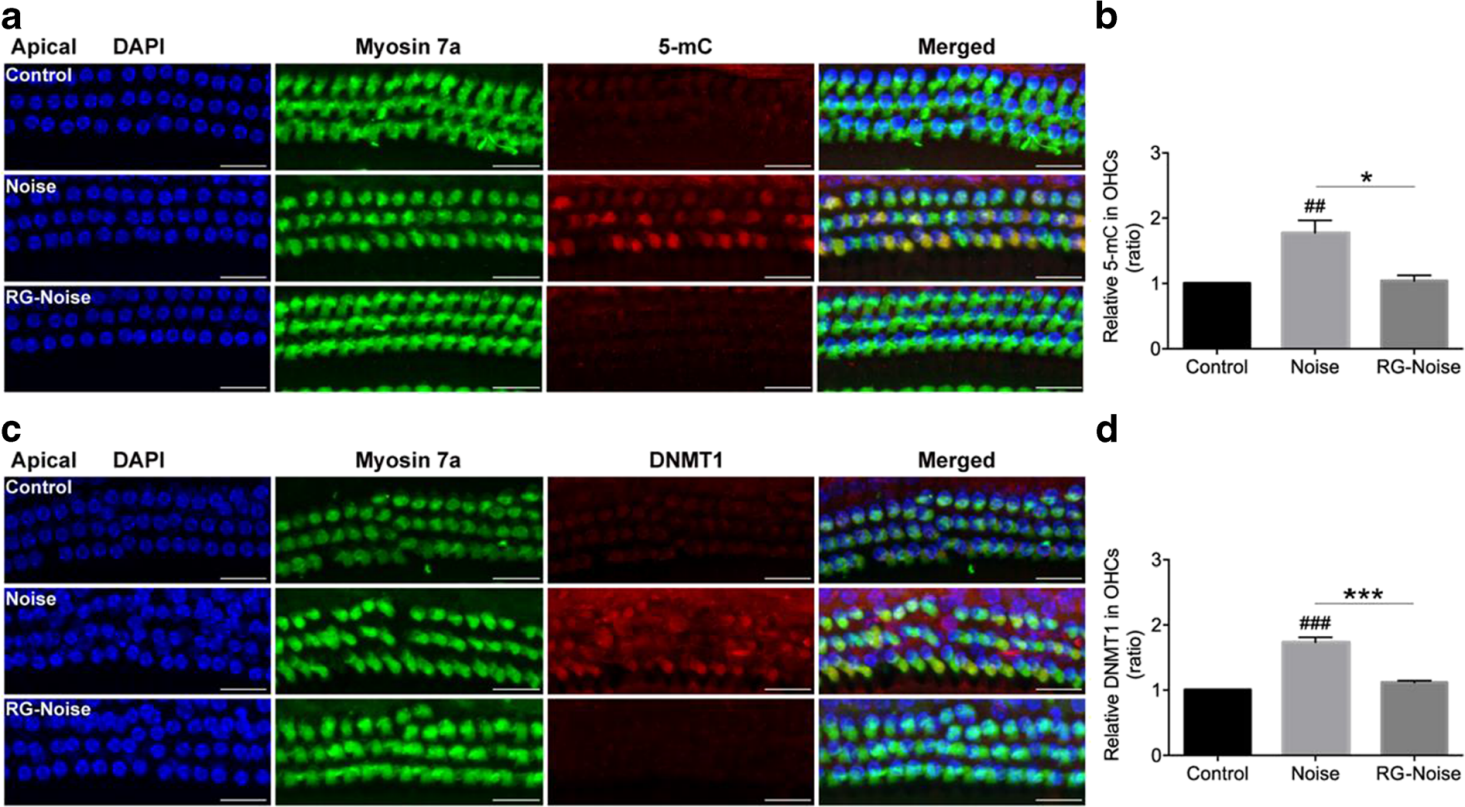
Immunofluorescence analyses of 5-mC and DNMT1 expression in OHCs. (a) Representative images showed an increase in immunoreactivity for 5-mC (red) in OHCs stained with myosin 7a (green) after noise exposure compared to controls without exposure. RG108 pretreatment significantly attenuated noise-induced 5-mC in OHCs. Images were taken from the apical turn. Scale bar =20 μm. (b) Quantification of 5-mC staining in OHCs confirmed a significant reduction with RG108 administration. *n* = 4 per group with one cochlea used per mouse. Data are mean ± SEM. ^##^*p* < 0.01 for noise vs. control; *p < 0.05 for noise vs. RG-Noise. (c) Representative images showed an increase in immunoreactivity for DNMT1 (red) in OHCs stained with myosin 7a (green) after noise exposure compared to controls without exposure. RG108 pretreatment significantly attenuated noise-induced DNMT1 in OHCs. Images were taken from the apical turn. Scale bar =20 μm. (d) Quantification of DNMT1 staining in OHCs confirmed a significant reduction with RG108 administration. *n* = 4 per group with one cochlea used per mouse. Data are mean ± SEM. ^###^*p* < 0.001 for noise vs. control; ***p < 0.001 for noise vs. RG-Noise

To determine whether RG108’s protective effects on NIHL may indeed be related to the inhibition of DNA methylation in mouse cochlear HCs, we evaluated DNA methylation levels in mouse cochlear specimens by 5-methylcytosine immunohistochemistry analysis. The results indicated that noise exposure led to DNA hypermethylation within the nuclei of outer hair cells compared with the unexposed group, while RG108 pretreatment reduced this noise-induced DNA hypermethylation (Fig. 7a, b). Furthermore, we analyzed global DNMT1 levels in outer hair cells by immunostaining, and DNMT1 amounts were overtly increased after noise exposure (Fig. 7c, d), in accordance with the DNA hypermethylation level of the genome. RG108 pretreatment significantly reduced noise-induced DNMT1 level increase (Fig. 7c, d). These results suggested DNA hypermethylation as an important mediator affecting noise-induced HC apoptosis.

**Fig. 8:**
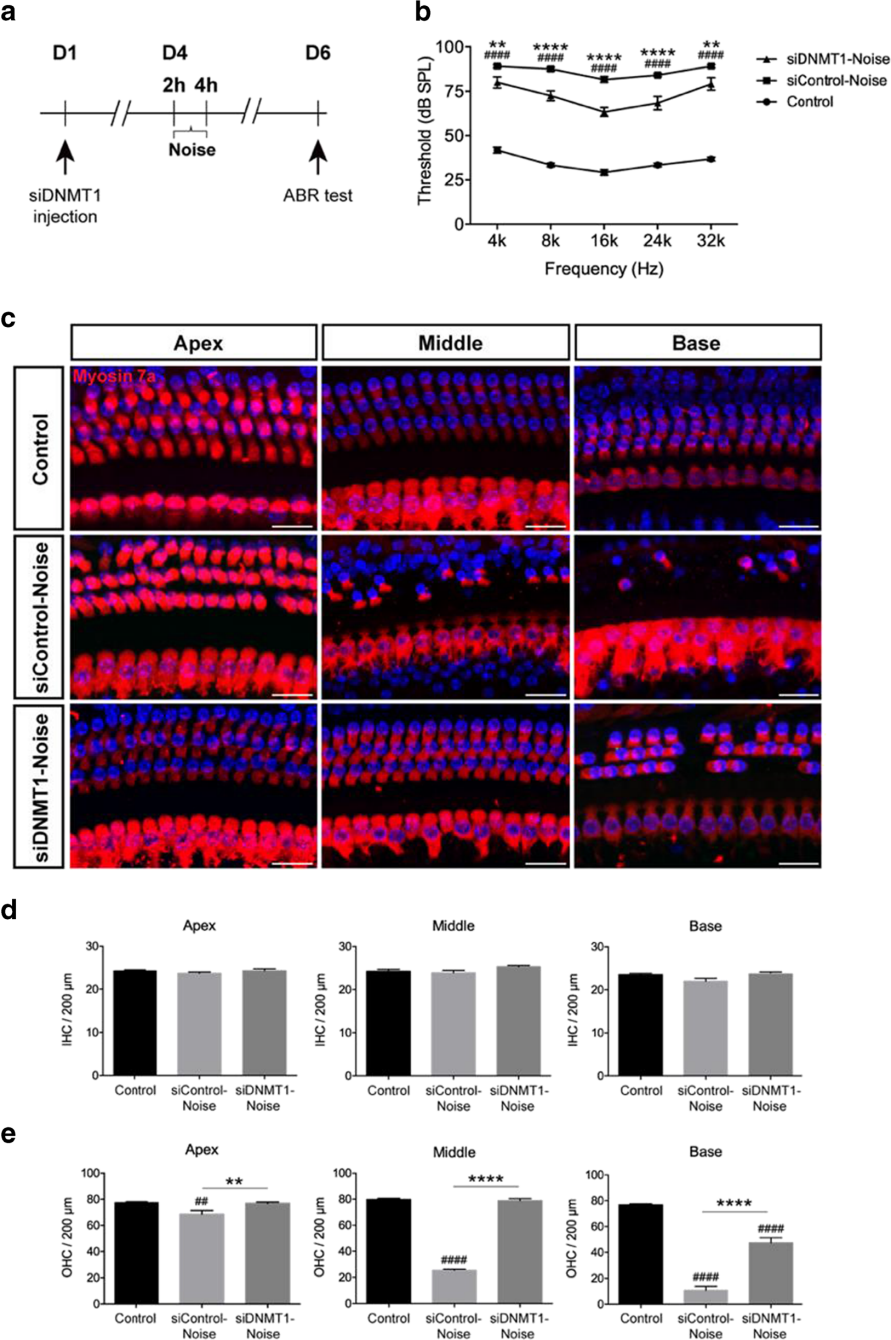
Evaluation of protective effect of siDNMT1 on noise-induced hearing loss. (a) Experimental schedule. Mice pre-injected with siControl or siDNMT1 were exposed to 120-dB noise for 2 h, and the ABR thresholds were measured at 4, 8, 16, 24, and 32 kHz at 2 days after noise exposure. (b) Noise exposure caused elevation of ABR thresholds across tested frequencies, while pretreatment with siDNMT1 significantly attenuated the noise-induced ABR threshold shifts at each tested frequency. *n* = 6 mice for each condition. Data are mean ± SEM. Statistical analysis was performed using a two-way ANOVA, ^####^*p* < 0.0001 for siControl-noise vs. control, **p < 0.01 and ****p < 0.0001 for siControl-noise vs. siDNMT1-Noise. (c) Representative images of myosin 7a staining of cochlear turns from different groups. Scale bar = 20 μm. (d, e) Quantitative analysis of noise-induced losses of IHC (d) and OHC (e) along the cochlear explants. Total IHC or OHC count per 200 μm was compared among no noise (control), siControl-noise, and siDNMT1-Noise groups. A significant loss of OHC was observed at the both base and middle turns in siControl noise-exposed mice compared with control mice. In contrast, OHC loss in the siDNMT1 pretreatment group was significantly lower than that in the siControl-noise group. No significant differences were observed in IHCs loss among the groups. *n* = 6 mice for each condition. Data are mean ± SEM. Statistical analysis was performed using a one-way ANOVA, ^##^*p* < 0.01, ^####^*p* < 0.0001 for siControl-noise vs. control; **p < 0.01 and ****p < 0.0001 for siControl-noise vs. siDNMT1-noise

We next evaluated the effect of DNMT1 on NIHL by siRNA-silencing. DNMT1 or siControl (scrambled siRNA) was administered via the round window in the mouse middle ear by post-auricular microinjection (Oishi et al. 2013). Treatment with a 0.6 μg dose of siDNMT1 significantly reduced fluorescent signals for DNMT1 in OHCs 3 days following administration compared with the siControl group (Supplementary Figure S2). The animals were exposed to the noise 3 days post-siRNA injections. Administration of siDNMT1 remarkably reduced noise-induced auditory threshold shifts at all frequencies (*n*=6, two-way ANOVA; Fig. 8a, b) at 2 days after noise exposure. Consistent with ABR measurements, myosin 7a signals in the cochlear epithelium reflected decreased OHC loss in both basal and middle cochlear regions in the siDNMT1 group compared with the siControl group (*n*=6, two-way ANOVA; Fig. 8d, e).

**Fig. 9:**
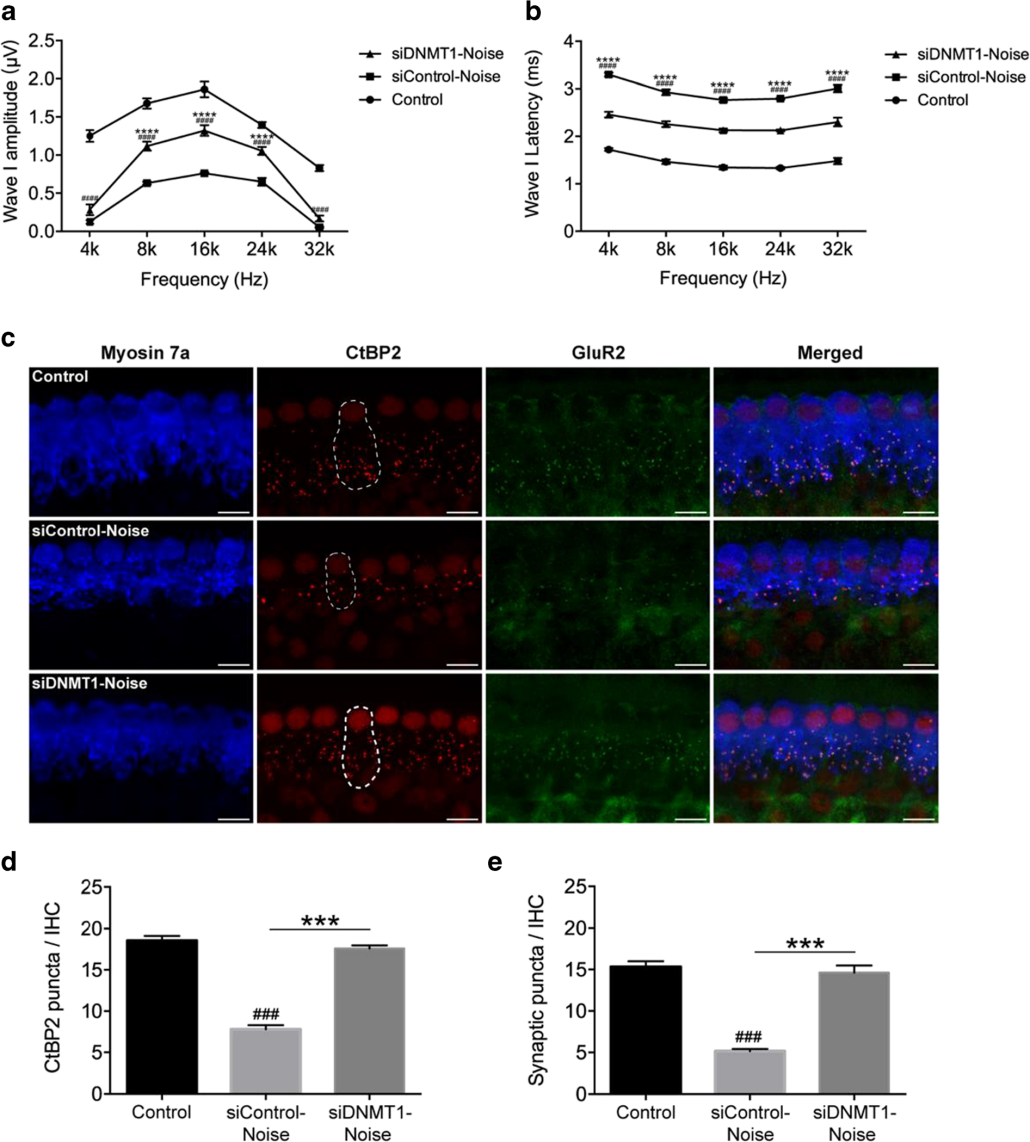
Evaluation of protective effect of siDNMT1 on noise-induced losses of synapses. (a) ABR wave I amplitudes in siDNMT1 pretreated mice were significantly increased compared to the noise exposed mice. (b) Threshold adjusted ABR wave I latency measurements demonstrated that siDNMT1 pretreatment prevented the increase of wave I latency induced by noise when tested 2 days after the noise exposure. *n* = 6 mice for each condition. Data are mean ± SEM. Statistical analysis was performed using a two-way ANOVA, ^####^*p* < 0.0001 for siControl-noise vs. control, ****p < 0.0001 for siControl-noise vs. siDNMT1-noise. (c) Representative confocal images of immunolabeling for presynaptic ribbons (CtBP2-red), postsynaptic receptor patches (GluR2-green), and IHCs (myosin 7a-blue) in the middle turn corresponding to 16 kHz in each group. The white dash lines indicate the outline of IHCs. Scale bar = 10 μm. (d, e) Quantitative data showed that noise reduced, and siDNMT1 pretreatment increased, the number of CtBP2-immunolabeled ribbons (d) and paired ribbon puncta, defined as juxtaposed CtBP2- and GluR2-positive puncta (e) at 16 kHz cochlear region; *n* = 24 IHCs from four cochleae of each group. Data are mean ± SEM. ^###^*p* < 0.001 for siControl-noise vs. control, ***p < 0.001 for siControl-noise vs. siDNMT1-noise

**Fig. 10:**
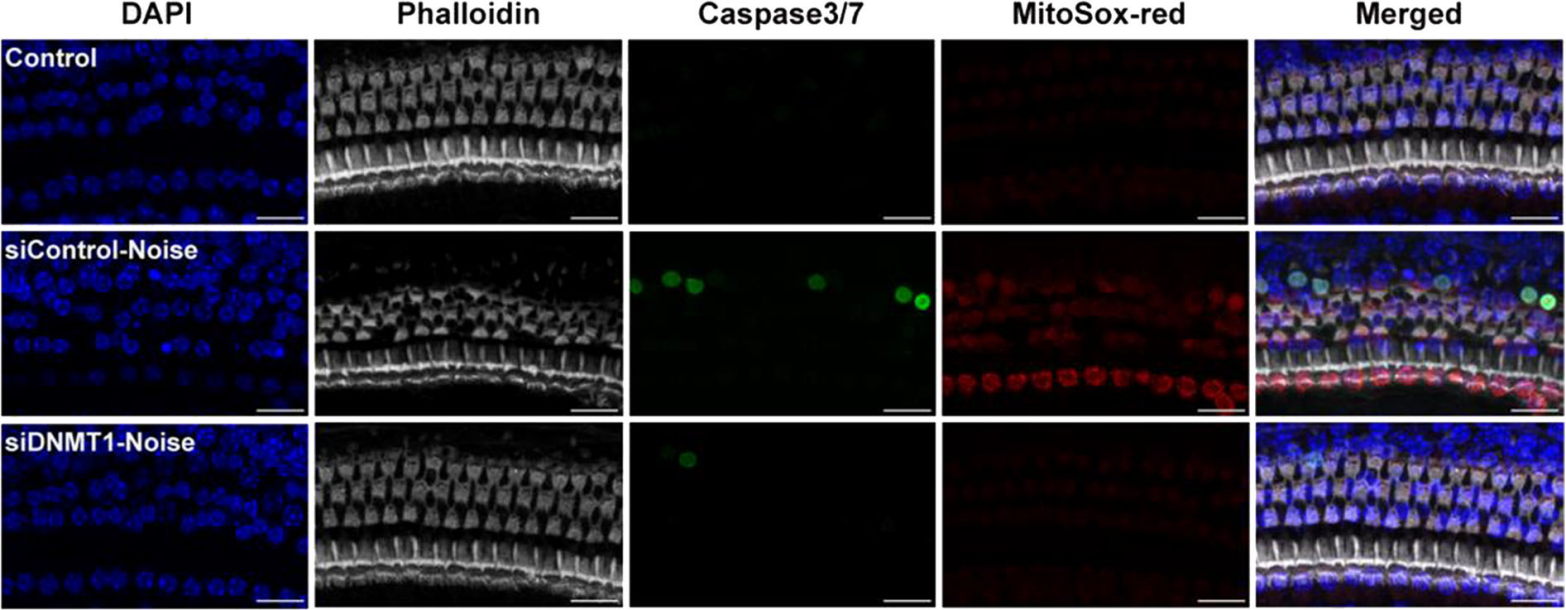
Detection of apoptotic cell death and mitochondrial ROS accumulation in the cochlea. Representative images showed an increase in immunoreactivity for caspase 3/7 (green) and MitoSox-red (red) in OHCs stained with phalloidin (white) after noise exposure compared to controls without exposure. Pretreatment with siDNMT1 significantly attenuated noise-induced caspase 3/7 and MitoSox-red in OHCs. Images were taken from the apical turn; Scale bar = 20 μm

We next determined whether DNMT1 blockade could attenuate noise-associated loss of IHC synaptic ribbons. To this end, ABR wave I amplitudes and latencies were measured, and ribbon synapses were counted 2 days after noise exposure. As depicted in Fig. 9a, siDNMT1 treatment prevented wave I amplitude reduction and latency increase associated with noise. In agreement with the results of RG108 treatment, siDNMT1 treatment significantly prevented noise-induced loss of CtBP2-labeled presynaptic ribbons and functional synapse complexes (CtBP2 + GluR2) on IHCs in the middle region (Fig. 9c–e). We further detected alterations of apoptosis and mitochondrial reactive oxygen species (ROS) under noise conditions versus normal control animals by caspase 3/7 and MitoSox-red staining. Administration of siDNMT1 starkly reduced noise-associated increases of caspase 3/7 and MitoSox-red signals in OHCs compared with the siControl group (Fig. 10), indicating that mitochondria-dependent apoptosis might contribute to the protective effect of DNMT1 blockage on NIHL.

## Discussion

Here, we demonstrated that noise exposure increased immunoreactivity of DNMT1 in the cochlea, inducing HC and cochlear synaptic ribbon losses and subsequent NIHL. DNMT1 suppression by pretreatment with DNMT1 siRNA or the administration of RG108, a selective DNMT1 inhibitor, significantly protected against noise-associated OHC and IHC synaptic ribbon losses and attenuated NIHL in adult mice, suggesting epigenetic modifications are responsible for hearing loss and may provide a good candidate for future therapy aimed at NIHL.

Recently, many studies using cell and animal models of hearing loss have identified dynamic patterns of several epigenetic modifications, implying that epigenetic dysregulation might have a correlation with the altered gene transcription observed in hearing loss (Chen et al. 2009; Chen et al. 2016; Wang et al. 2013). There have been many experimentally used epigenetic drugs that target posttranslational histone modifications, which contribute to the pathogenesis of hearing loss. A previous study by our research group reported that neomycin-induced elevations of histone methyltransferase G9a and H3K9me2 amounts are associated with aminoglycoside-induced hair cell loss, and pharmacological targeting of G9a with BIX 01294 could prevent damage to inner ear hair cells (Yu et al. 2013). Delivery of G9a siRNA *in vivo* 72 h prior to noise exposure also significantly reduced ABR threshold shifts and ameliorated noise-induced permanent hearing loss (Xiong et al. 2019). Additionally, a histone deacetylase inhibitor, suberoylanilide hydroxamic acid, was shown to protect outer hair cells from noise-induced damage, hence, raising the possibility that epigenetic regulation may be a potential therapeutic strategy to prevent hearing loss (Wen et al. 2015).

DNA methylation, another well-studied and important epigenetic modification that regulates gene expression without changing the DNA sequence, is implicated in numerous biological processes (Smith and Meissner 2013). Indeed, abnormal DNA hypermethylation and hypomethylation are closely related to pathological conditions, particularly cancer (Jones and Baylin 2007). There are three functional DNMTs: DNMT1, DNMT3A, and DNMT3B. DNMT1 represents a maintenance enzyme, while DNMT3A and DNMT3B exhibit predominant *de novo* methyltransferase activities (Auclair and Weber 2012; Bird 2002). It has been reported that the DNMT suppressor 5-aza-2′-deoxycytidine (5-aza-CdR), a cytosine derivative that is incorporated into genomic DNA and irreversibly interacts with DNMTs, demethylates DNA in the whole genome of MUCs and significantly upregulates epithelial and hair cell genes (Zhou and Hu 2015). A recent study (Deng et al. 2019) showed that treatment of MUCs with 5-aza starkly decreased DNMT1 protein amounts and DNMT activity, upregulating *Sox2* (Kiernan et al. 2005), a transcription factor critical for the formation of hair cells and supporting cells in mammalians’ inner ear, and *Lfng* (Pujades et al. 2006), which is implicated in early sensory organ development, inducing hair cell differentiation. These findings highlight DNA methylation as an important mechanism underpinning epigenetic modulation of genes in inner ear stem cells, with a critical function in stem cell fate determination. However, the involvement of DNA methylation in the pathogenesis of NIHL remains undefined.

Here, by assessing DNA methylation in cochlear specimens from mice, we found that noise exposure increased DNA 5-mC and DNMT1 amounts in mouse HCs, suggesting a potential link between aberrant regulation of DNA methylation and NIHL. RG108 is a novel, non-nucleoside inhibitor with lower cytotoxicity or genotoxicity compared with other DNMT inhibitors, which works primarily by interacting with the catalytic domain of DNMT1 to inhibit DNA methylation (Brueckner et al. 2005). A recent study showed that DNMT is upregulated in motor neurons of adult mice during apoptosis, and treatment with the DNMT inhibitor RG108 significantly protected motor neurons from apoptosis in cell and animal models (Chestnut et al. 2011). Thus, in the present work, mice were administered RG108 2 h before noise exposure, and auditory brainstem responses induced by tone burst upon noise exposure were evaluated. Compared with ABR thresholds in the noise group, the animals administered RG108 had remarkably reduced values at 2 days after noise exposure. Further counting of hair cells by myosin 7a immunoreactivity from apex to base along the whole cochlear epithelium suggested that noise exposure increased outer hair cell loss along an apex-to-base gradient. After pretreatment with RG108, outer hair cell loss was starkly reduced, especially in both basal and middle segments of the epithelium, compared with the noise only group. These results corroborated ABR functional data. Furthermore, reducing DNMT activity by injecting siDNMT1 prior to noise exposure mimicked RG108 administration, attenuating noise-associated OHC loss and the resulting hearing impairment, which indicates that alleviation of noise-related OHC loss and NIHL by RG108 treatment indeed relies on DNMT1 suppression. As demonstrated above, phalloidin staining showed overt morphological alterations with OHC loss following noise exposure, while RG108 treatment protected OHCs from noise-related damage. Further examination of cochlear specimens from mice treated with noise showed that IHCs were almost unaltered along the whole cochlear spiral but the numbers of IHC ribbon synapses and auditory nerves were significantly reduced (Furman et al. 2013). Beside hair cell protection, RG108 treatment decreased noise-associated IHC synaptic ribbon loss, hence, preventing ABR wave I amplitude reduction and wave I latency increase. The present quantitative analyses of IHC synapses, ABR wave I amplitude and latency, and cochlear nerve terminals corroborate data obtained after siDNMT1 administration, indicating DNMT1 as a critical epigenetic mediator of cochlear synaptopathy.

The TUNEL assay showing increased TUNEL-positive cells in the noise group indicated that hair cell loss after noise exposure resulted from apoptosis. The DNA methylation inhibitor RG108 significantly inhibited noise-induced apoptosis in the mouse cochleae. Knockdown of DNMT1 showed similar effects as RG108 treatment in terms of reduced caspase 3/7 immunoreactivity in cochlear hair cells. Emerging evidence indicates that increased oxidative damage can be caused by noise exposure and promotes subsequent NIHL progression (Henderson et al. 2006). In support of this notion, administration of antioxidants, including glutathione (Ohinata et al. 2000), acetyl-l-carnitine, and N-l-acetylcysteine (Kopke et al. 2005), before noise exposure was shown to attenuate noise-induced hearing loss; conversely, impairment of antioxidant defense pathways, e.g., glutathione peroxidase inactivation (Ohlemiller et al. 2000), was revealed to increase noise-induced cochlear injury in mice. Therefore, strengthening cochlea cell resistance toward excessive oxidative stress for maintaining intact cochlear structure might represent an effective approach in alleviating NIHL progression. In the present study, RG108 treatment could suppress noise-induced oxidative damage, as evidenced by the expression of 3-NT, a marker of oxidative stress (Yamashita et al. 2004). The increased levels of 3-NT in OHCs upon noise exposure were markedly attenuated by treatment with RG108. These data are consistent with previous observations reported for human bone marrow mesenchymal stromal cells using RG108 (Oh et al. 2015), indicating that RG108 could alleviate cellular damage due to oxidative stress. We also observed that application of siDNMT1 prior to noise exposure significantly reduced mitochondrial ROS, suggesting that suppressing oxidative damage associated with noise is involved in the otoprotective effect of DNMT1 inhibition. Considering that an increasing number of drugs prevent hearing loss induced by noise, e.g., antioxidants (Wu et al. 2010), neurotrophic factors, anti-inflammatory drugs (Kyle et al. 2015), and calcium-channel blockers (Minami et al. 2004), investigating whether the otoprotective effects of RG108 could be strengthened by combination with these therapeutic agents may be a useful approach. Although apoptosis and oxidative stress were alleviated in cochleae from RG108-treated mice exposed to noise, there were still several limitations in this study. For instance, it is not clear whether this effect is due to a direct modulation or not. The detailed and precise mechanisms by which DNMT1 inhibition protects from OHC and IHC synapse losses after noise damage need further investigation. Based on the mechanism of RG108 as a DNMT inhibitor, it is possible that it exhibits antiapoptotic and antioxidant effects by upregulating a great number of genes via epigenetic mechanisms. Therefore, global genomic analysis of RG108-treated hair cells may provide novel clues to understanding the underlying mechanisms of RG108.

Systemic administration is commonly prescribed for NIHL treatment, and one of the most utilized routes in mouse studies is intraperitoneal (IP) administration (Al Shoyaib et al. 2019). This easy-to-perform technique is fast with low impact on animals. However, the efficacy of systemic administration for treating inner ear diseases is frequently limited by the blood–labyrinth barrier. Local administration allows sufficient amounts of therapeutics to access the inner ear while bypassing the blood-labyrinth barrier with minimal systemic side effects. Several local surgical injections have been developed to access the inner ear. Round window membrane (RWM) injection is the most common delivery technique in which the therapeutic agent is directly administered into the perilymphatic space (Akil et al. 2012; Dai et al. 2017; Landegger et al. 2017; Pan et al. 2017; Yoshimura et al. 2018). This injection is clinically feasible, and cochlear implantation in humans routinely relies on surgical electrode insertion through the RWM (Gantz and Tyler 1985; Nguyen et al. 2016). Here, we assessed the therapeutic effects of RG108 given systemically and siDNMT1 administered via RWM in the mouse, respectively, and both promoted hearing recovery as measured by ABRs. However, a limitation of this treatment is that RWM injections often lead to fluid leakage and induce a basal–apical gradient, as seen with injected fluorescein (Akil et al. 2019; Plontke et al. 2016). Currently, more effective delivery routes for inner ear treatment are needed. Posterior semicircular canal (PSCC) represents another attractive site for inner ear injections, because it is anatomically easy to locate, which simplifies the surgical approach and decreases the chance of middle ear damage by surgery (Isgrig and Chien 2018; Kawamoto et al. 2001; Suzuki et al. 2017). PSCC injection has been reported in many studies in cochlear and vestibular hair cell transduction (Okada et al. 2012; Suzuki et al. 2017). Yoshimura et al. described RWM injection with semicircular canal fenestration in PSCC as a safe technique for cochlear gene therapy (Yoshimura et al. 2018). A study by Talaei et al. reported that PSCC injection induces higher levels of dye delivery within neonatal and adult mice cochleae compared with RWM injections (Talaei et al. 2019). Additionally, a technical shortcoming of PSCC injection is that it remains uncertain whether the injected therapeutic agent is administered into the endolymph or perilymph (Kawamoto et al. 2001). It would be useful in future studies to evaluate the therapeutic effect of DNA methylation inhibition in NIHL using different administration routes such as RWM and PSCC injections.

## Conclusions

Overall, these data firstly established that RG108, a non-cytotoxic demethylating agent, has the therapeutic potential to prevent and treat NIHL by blockade of DNMT activity. Indeed, RG108 administration alleviated noise-associated abnormal DNA hypermethylation and oxidative stress in cochlear HCs and reduced HC and synaptic ribbon losses. Future work should elucidate comprehensive molecular and cellular mechanisms underpinning the role of RG108, to further improve its effectiveness as a promising otoprotective drug.

## Supplementary Information

ESM 1 (DOCX 4379 kb)

## Data Availability

The datasets during and/or analyzed during the current study available from the corresponding author on reasonable request.

## Abbreviations

3-NT: 3-nitrotyrosine
4-HNE: 4-hydroxynonenal
5-aza-CdR: 5-aza-2′-deoxycytidine
ABR: auditory brainstem responses
CtBP2: C-terminal binding protein 2
DMSO: dimethyl sulfoxide
DNMT: DNA methyltransferase
DNMT1: DNA methyltransferase-1
EDTA: ethylenediaminetetraacetate
HC: hair cell
IHCs: inner hair cells
IP: intraperitoneal
MUCs: mouse utricle sensory epithelia-derived progenitor cells
NIHL: noise-induced hearing loss
NF: neurofilament
OCT: optimal cutting temperature compound
OC: organ of Corti
OHC: outer hair cells
PFA: paraformaldehyde
RNS: reactive nitrogen species
ROS: reactive oxygen species
SEM: scanning electron microscopy
SGN: spiral ganglion neurons
SL: spiral ligament
TUNEL: terminal deoxynucleotidyl transferase-mediated dUTP nick-end labeling
