# Supplementary material for: The DNA methylation inhibitor RG108 protects against noise-induced hearing loss
Source: Cell Biol Toxicol. 2021 Mar 15;37(5):751–71. doi: 10.1007/s10565-021-09596-y (PMC8490244; doi:10.1007/s10565-021-09596-y)
Supplement: Supplementary file 1 — (DOCX 4379 kb) [file 10565_2021_9596_MOESM1_ESM.docx]

**Supplemental figure legends**


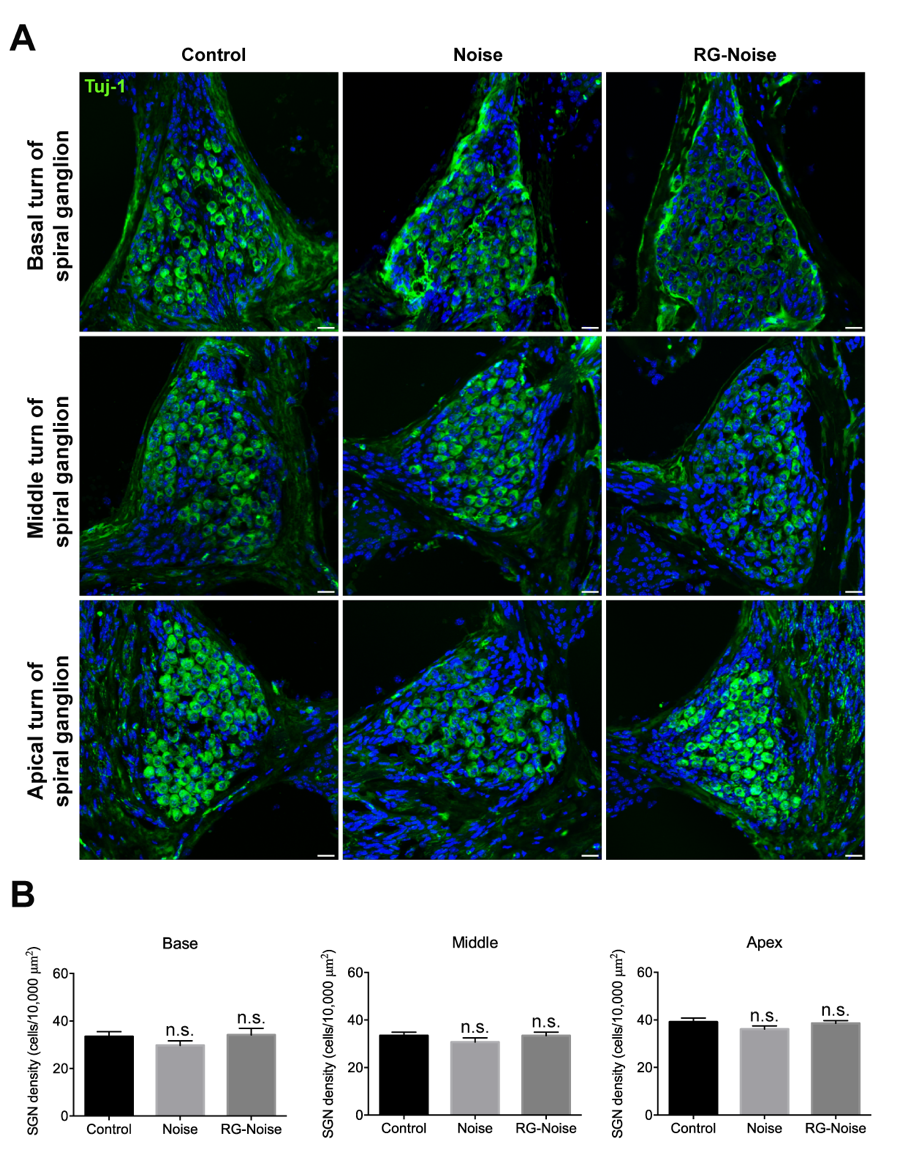


**Supplemental Figure 1.** Detection of spiral ganglion neurons (SGNs) in the cochlea. (**A**) Representative images of Tuj-1 staining of spiral ganglion neurons (SGNs) at apical, middle, and basal turns of cochlea from different groups at 2 days after noise exposure. Scale bar = 20 μm. (**B**) Density of spiral ganglion neurons at basal, middle, and apical turns of cochlea were measured in the inner ear sections. n.s., nonsignificant.


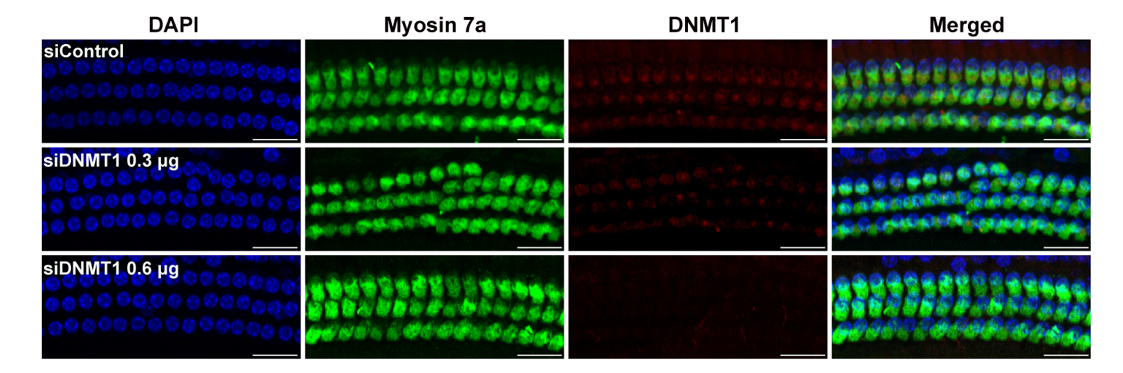


**Supplemental Figure 2.** Immunofluorescence analyses of DNMT1 expression in OHCs. Representative images showed a decrease in immunoreactivity for DNMT1 (red) in OHCs stained with myosin 7a (green) after delivery of siDNMT1 compared to siControl. 0.6 μg dose of DNMT1 significantly attenuated expression of DNMT1 in OHCs. Scale bar =20 µm.
